# Welfare implications of injuries and deformities in wild fish

**DOI:** 10.1017/awf.2026.10083

**Published:** 2026-04-15

**Authors:** Simon Eckerström Liedholm

**Affiliations:** Wild Animal Initiative, Minneapolis, MN 55437, USA

**Keywords:** Animal welfare, disease, mortality rate, ontogeny, teratology, welfare assessment

## Abstract

External injuries and morphological deformities may serve as useful indicators when assessing the welfare of wild animals, as they can be easily observed, be scored in a non-disruptive manner, and likely correlate with reductions in welfare in many contexts. However, the welfare effects of injuries and deformities have so far been mostly examined for animals in captivity. In contrast, the many fish living in the wild have received considerably less attention, especially in relation to naturally occurring causes, such as parasitism, predation attempts, and intra-specific conflict. Here, I attempt to quantify the prevalence of injuries and deformities in wild fish by conducting a targeted review of six relevant journals, and suggest areas where future research would be particularly useful. The results indicate that both anthropogenic and non-anthropogenic factors can cause injuries and deformities in wild fish, and that many of the focal species (i.e. the species studied in the reviewed papers) are closely related. The average prevalence of injuries and/or deformities was 15% across studies. Despite the existence of potential confounding factors (e.g. a selection bias in terms of focal populations and species), these results highlight the potential importance of injuries and deformities as determinants and indicators of fish welfare in the wild.

## Introduction

Both injuries and deformities are commonly observed across animal species (Sokos *et al.* 2018; Rennolds & Bely 2023). Injuries, as defined in this review, correspond to any physical damage to biological tissues, whether induced mechanically (Rennolds & Bely 2023), by environmental factors (such as wounds caused by toxins or heat), or as a manifestation of a disease (e.g. skin lesions or ulcerations). It should be noted that this is a broad definition of ‘injury’ that is akin to measuring external physical damage, and does not separate out the sources of such damage (see e.g. Breitmeyer *et al.* 2025). The aetiology of external tissue damage can however be difficult to determine. This, and the fact that several of the studies reviewed do not diagnose causes or perform histopathology, mean that for simplicity all such tissue damage is grouped here under ‘injury’. When animals survive injuries that significantly affect their morphology, injuries may lead to deformities (Noble *et al.* 2012). Deformities are here defined as persistent morphological abnormalities, whether acquired through injury (Eissa *et al.* 2021) or congenital in origin, that can reduce the ability of juveniles to reach adulthood (Sokos *et al.* 2018). Despite intrinsic differences (e.g. chronicity and healing mechanisms), both injuries and deformities can negatively impact the welfare of animals, for instance by generating pain or by inducing hunger through limited access to resources (Gregory 2004; Mellor *et al.* 2020; Rennolds & Bely 2023). However, the severity and duration of welfare impacts due to a given injury or deformity in fish is likely lower than for terrestrial animals; many species of fish are able to regenerate tissues (Poss *et al.* 2002) and benefit from buoyancy and structural support in water. As these external markers can theoretically be observed in the wild without disturbing animals, they may be of great use for wild animal welfare assessments by increasing the feasibility of assessing welfare (Browning *et al.* 2024, in prep).

If the ultimate objective of wild animal welfare research (Soryl *et al.* 2021) is to improve the welfare of as many individuals as possible (John & Sebo 2020), welfare indicators should be applicable to species that are highly numerous. Among vertebrates, fish are more numerous than non-fish in terms of the number of individuals alive at any point in time (Bar-On *et al.* 2018). Moreover, they are also at least plausibly sentient (de Mori & Normando 2019) and thus capable of having welfare. However, research on the welfare effects of injuries and deformities (Huntingford *et al.* 2006; Branson & Turnbull 2008) has so far mostly focused on farmed/captive fish (Noble *et al.* 2012). As in other animals, injuries and deformities in fish have often been associated with significant reductions in their welfare (Huntingford *et al.* 2006; Branson & Turnbull 2008), due to pain (Gregory 2004) and possible indirect consequences, such as an increased disease risk, reduced feeding success, impaired locomotion, or diminished competitive ability (Ángeles Esteban 2012).

Even though injuries and deformities may be useful to monitor and improve the welfare of captive fish (Noble *et al.* 2012), they may be even more useful in the welfare assessment of wild fish, as they are more numerous and challenging to monitor. Moreover, wild fish may not be affected by injuries and deformities in the same ways as their captive counterparts. For instance, fin erosion (Turnbull *et al.* 1998; Latremouille 2003) is expected to occur more frequently in captivity, to the point that it can be used to distinguish hatchery-reared salmonids from their wild counterparts (see, e.g. McDonald & Hume 1984). In contrast, wild fish populations are expected to be affected by many natural factors that cause injuries and deformities, such as predation attempts or parasitic infections (MacKenzie *et al.* 1998; Bergstedt *et al.* 2001; Papastamatiou *et al.* 2010; Sánchez *et al.* 2018), pathogens (Smith 2019), or hypoxia and high temperature causing deformities during development (Purcell *et al.* 1990; Shang & Wu 2004; Ytteborg *et al.* 2010; Ma *et al.* 2016). Wild fish are also likely to suffer from injuries and deformities due to certain anthropogenic factors, such as exposure to pollutants (e.g. heavy metals or organochlorine pesticides; Kingsford & Gray 1996; Mebane *et al.* 2003; Hassell *et al.* 2020) or large fishing operations. Such fishing operations may decrease the welfare not only of the fish retained for food and feed (Metcalfe 2009; Veldhuizen *et al.* 2018; Breen *et al.* 2020), but also of those that are not targeted and therefore released. Released individuals may sustain injuries that substantially affect their near-term or long-term survival (Broadhurst *et al.* 2006), potentially by increasing infection and predation risk (Broadhurst *et al.* 2006; Raby *et al.* 2014). Even if they survive, released individuals may still experience pain due to descaling, fin damage and rapid pressure changes during capture (Veldhuizen *et al.* 2018). Given that the global bycatch tonnage is around 40% of the total global catch (Davies *et al.* 2009), and the total number of fish caught annually is likely more than one trillion (10^12^) individuals (Mood & Brooke 2024), this might constitute one of the largest anthropogenic causes of injuries and subsequent suffering in wild fish (and potentially in wild vertebrates). Lastly, the welfare effects of injuries and deformities on juveniles is particularly important, given the large number of juveniles (Hill *et al.* 2021).

Predicting how multiple factors might affect welfare through injuries and deformities in wild fish can prove difficult, given that one factor might reinforce or attenuate the effect of other factors (see e.g. Bakke & Harris 1998). However, simply knowing the prevalence of injuries and deformities could help better understand the associated welfare burden for wild animals, even though this may be particularly challenging to estimate it in the wild, particularly for fish. For instance, because mortality rate is expected to be increased in individuals experiencing injuries and deformities (Nicola & Cordone 1973; Purcell *et al.* 1990; Hostetter *et al.* 2011), particularly in the early developmental stages (Hickey 1979), the data observed in a given population at a given time are expected to be effectively censored relative to the total number of animals affected at any point. This means that while the number of individuals affected by injuries or deformities at a particular point in time is accurately reflected, the total number of individuals that experience pain or suffering due to injuries or deformities *at any point* in their lives is likely underestimated when examining prevalence at a given time-point. Accounting for the increased mortality due to injury or deformity is therefore likely to provide better estimates of the total number of individuals that ever experienced injuries and/or deformities in their lives (see e.g. Schneider *et al.* 1996; Hostetter *et al.* 2012). Ultimately, combining this number with the duration of time spent experiencing injuries and deformities, and the severity of the reduction in welfare due to injuries and deformities, could provide estimates of welfare loss due to injuries and deformities at the population level (assuming the approach to aggregating welfare is similar to the estimation of quality-adjusted life years (QALYs); see, e.g. Hecht 2021). Injuries and deformities that severely affect basic functions, such as breathing (Branson & Turnbull 2008) or feeding (Purcell *et al.* 1990), are likely to have strong effects on both welfare and survival, while minor injuries or deformities may not affect welfare and survival as much but may last longer and occur more frequently. The mortality rate itself, such as during mass mortality events (La & Cooke 2011; Phelps *et al.* 2019), may also be a decent indicator of poor welfare, if moribund fish suffer a great deal before death (Ellis *et al.* 2012; Hecht 2021).

To shed a degree of light on the prevalence of injuries and deformities and their welfare effects in wild fish, I conducted a targeted review of papers in a select number of journals assumed to be representative of the field of fish biology. To characterise the state of the research on injuries and deformities in wild fish, I recorded the species name, the age-class of sampled individuals, and the causal factor (anthropogenic vs non-anthropogenic) under consideration in the selected studies. To evaluate the feasibility of monitoring injuries and deformities for wild fish welfare assessments, I also recorded the collection method to assess how easily injuries and deformities can be measured, and the level of disturbance/disruption to the study subjects. Altogether, the results of this review are expected to provide the first picture of the extent to which the welfare of wild fish is affected by injuries and deformities.

## Materials and methods

### Search procedure

The six journals included in this review are presented in Table 1. These journals were selected because they are well-established outlets for research on fish health, aquatic toxicology, and fisheries science, and were therefore considered likely to contain a meaningful fraction of all studies reporting on injuries and deformities in wild fish. While this selection does not cover all relevant journals, it was designed to capture a representative cross-section containing the most relevant journals. The data collection was performed by searching and recording the first 100 search hits (sorted by the search functions based on relevance) for each of the three search sets (see below) across all available dates, by using the search tools available on the website of each journal. All searches were conducted during December 2023 and January 2024. The following three search sets were used: ["wild fish" AND “abnormalities”], ["wild fish" AND “deformities”], and ["wild fish" AND “injury”]. Boolean operators (“AND”) and searches for exact phrases were used where such functionality was available. The total number of unique search hits was 540 (see Table 1 for a breakdown by journal). A small fraction of search hits seemed not to pertain to any specific scientific research product (~1% of search hits were tables of content for journal volumes).

**Table 1.** The number of papers retained for each journal in review seeking prevalence of injuries and deformities in wild fish

Journal	Unique search hits	Retained papers
*Aquatic Toxicology*	158	1
*Canadian Journal of Fisheries and Aquatic Sciences*	61	8
*Journal of Fish Biology*	68	2
*Journal of Fish Diseases*	78	11
*Journal of the Marine Biological Association of the United Kingdom*	99	1
*Transactions of the American Fisheries Society*	76	7

To refine the initial list of articles in terms of relevance, I applied the following set of criteria sequentially, for which all statements had to be true: (1) An empirical study was described, where at least one part of the study described observational data; (2) At least one of the focal species in the study was a fish (i.e. non-tetrapod vertebrate); (3) At least one of the studied populations of fish were free-living and not confined by man-made structures (e.g. a pond), and could be assumed to have been free-living since their early development; (4) Observational frequency data were presented on injuries and/or deformities of these free-living fish, where such injuries and/or deformities could plausibly be considered detrimental to their welfare;(5) At least one of the different types of measured injuries and/or deformities in those free-living fish was visible externally with the naked eye.

Of the 540 unique search hits, 482 (482/540; about 89%) described empirical studies. Four hundred and four (404/482 about 84%) of those presented data on at least one species of fish. One hundred and sixty-two (162/404; about 40%) of those were studies that included free-living fish. Fifty-four (54/162; about 33%) of those presented observational frequency data on injuries and/or deformities in free-living fish. Thirty (30/54; about 56%) of those included observational frequency data where at least one of the types of measured injuries and/or deformities of the free-living fish was externally visible with the naked eye. Hence, about 6% (30/540) of the unique search hits were retained for further processing. The number of papers retained for each journal can be found in Table 1.

### Information extracted from retained studies

In the 30 studies that passed all the selection criteria, the following information was extracted: the species name(s); the age-class (non-sexually vs sexually mature individuals); the type of traits measured (injuries and/or deformities); the frequencies of the injuries and/or deformities; the presence or absence of appreciable human disturbance (explicitly mentioned or inferred); and the sampling method used. It should be noted that classifying human disturbance as a binary variable is a simplification, given that virtually all aquatic environments are affected by human activity, at least to some degree. Nevertheless, this classification was used as a practical way of distinguishing between sites with strong, direct, and localised anthropogenic impacts (e.g. industrial effluents or oil spills) and sites without such direct impacts. Among the retained studies, some focused on multiple species, presented data from both disturbed and undisturbed environments, or reported prevalence estimates for multiple traits. In those cases, data were extracted such that one estimate for every species-trait-disturbance combination was retained (see Supplementary file S2; Supplementary material), resulting in a total of 43 prevalence estimates. For each species in each study, percentages were averaged over study sites, year of sampling, and ages/developmental stages of fish, where these were reported separately in the study. To test whether the species included in this study constituted a random sample of fish species, a test of the phylogenetic signal (based on presence/absence in the current study) was conducted using a phylogeny of about 2,000 species of fish (Betancur-R *et al.* 2017), using the package ‘phylosignal’ (Keck *et al.* 2016) in R (R Core Team 2024). Here, the ‘trait’ for which the phylogenetic signal was assessed represents the combination of factors that influence whether a particular species is studied by humans or not. For the full set of species that passed all criteria in this study, see Figure 2 and Supplementary file S4 (Supplementary material).

Note that because of the heterogeneity of the methods used in the different studies and the non-comprehensive scope of this review, the results of this study could not be used for a meta-analytical approach and should therefore be interpreted with caution.

## Results

### Prevalence of injuries and deformities

Out of the 30 studies retained, 23 focused on injuries (many of which were related to diseases) and eleven focused on deformities (with four focusing on both injuries and deformities). The types of injuries and deformities that were recorded varied from general (e.g. whether the fish appeared normal or showed any signs of abnormalities) to specific (e.g. clinical signs of the Red Vent Syndrome; Noguera *et al.* 2015). Some studies focused on a single trait category, while others reported data on multiple separate traits (see Supplementary file S1; Supplementary material). Only two studies reported increased mortality rates associated with the presence or absence of injuries or deformities.

**Figure 1.**
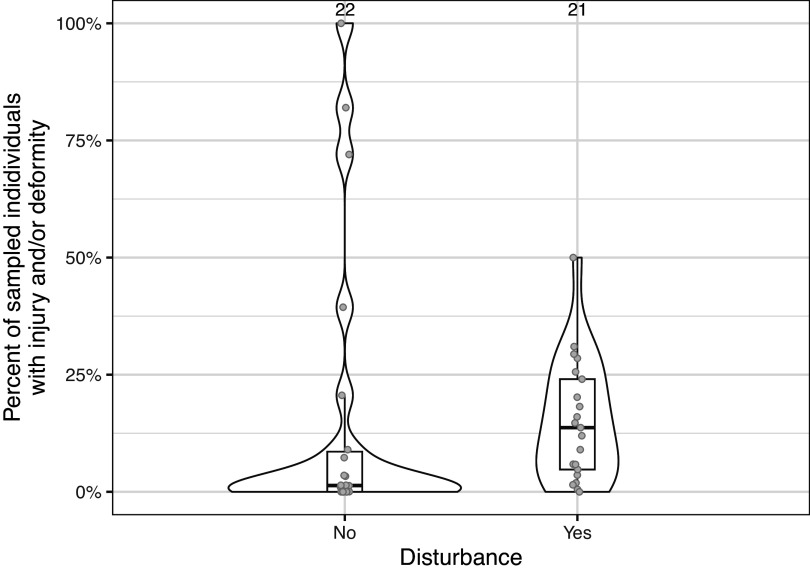
Percentage of sampled fish (43 estimates from 30 studies on a total of 22 species) with injury and/or deformity for disturbed vs undisturbed populations or sites; data from a review of 30 published articles on injuries and deformities in wild fish. Boxplots represent the median and the interquartile range (whiskers span data-points within 1.5 × the interquartile range). The code to recreate the plot can be found in Supplementary file S5 (see Supplementary material).

Based on the selected studies, it appears that the prevalence of injuries (mean [± SD] prevalence exclusively for injuries: 21.2 [± 26.9]; n = 27) and deformities (mean [± SD] prevalence exclusively for deformities: 6.8 [± 7.4]; n = 13) was highly variable across estimates (mean [± SD] prevalence for all data: 15.3 [± 22.9]; n = 43, see Supplementary file S5; Supplementary material). Disturbance levels seemed to differ mostly in terms of the spread and the shape of their respective distributions (see Figure 1; mean [± SD] prevalence in non-disturbed: 15.6 [± 29.8]; n = 22; mean [± SD] prevalence in disturbed: 15.1 [± 12.9]; n = 21). The sample sizes for estimating the prevalence also varied considerably across estimates (mean [± SD]: 4,656 [± 14,307], range = 4 to 81,733; n = 37).

### Species and age class representation

**Figure 2.**
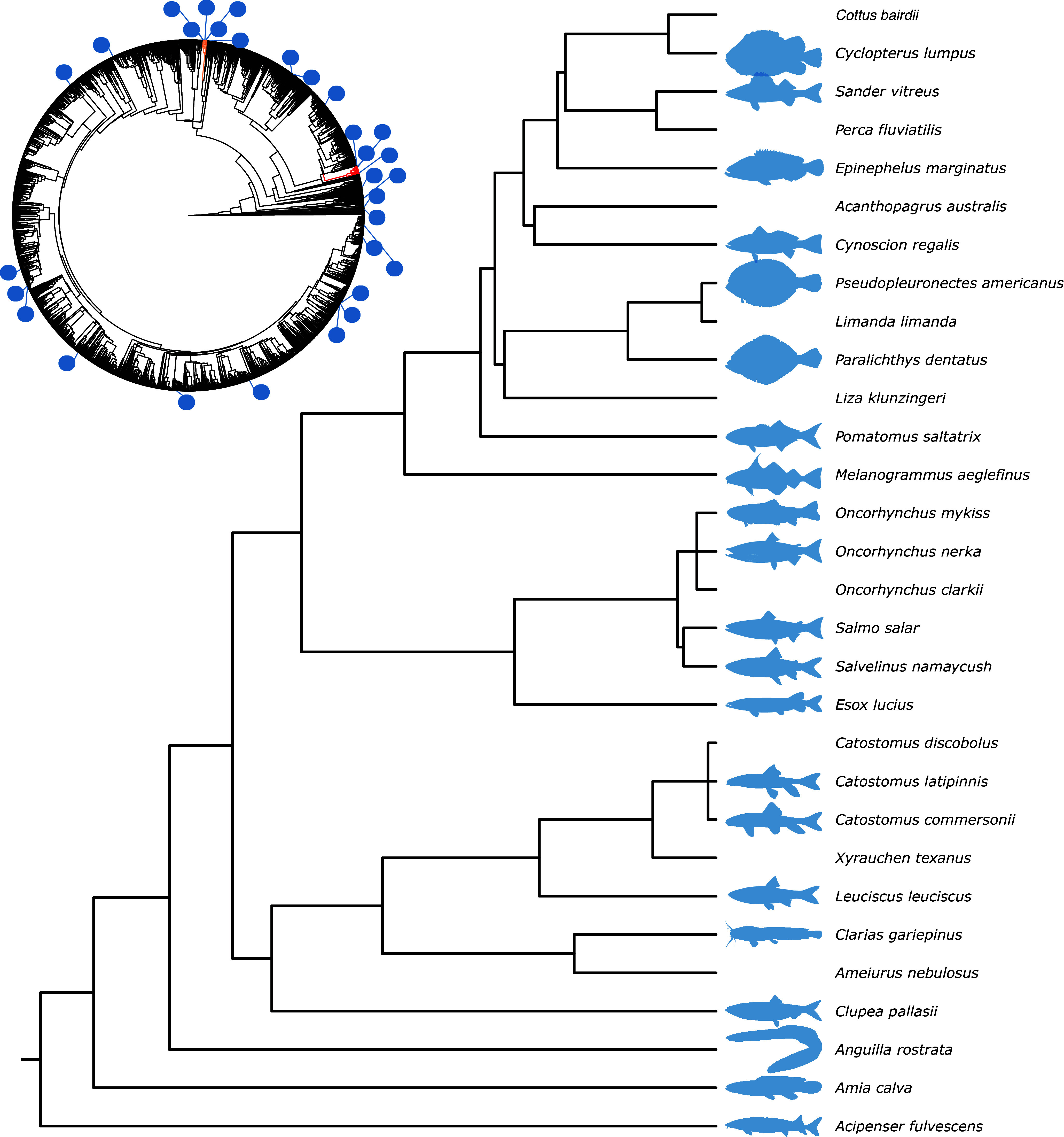
Phylogenetic tree of the fish species included in the review, showing their evolutionary relationships. The full tree and the pruned tree are modified versions of the complete tree published by Betancur-R *et al.* (2017), with additional species grafted onto it. The branches leading to the Salmonidae and the Catostomidae families have been coloured orange and red, respectively, in the upper-left phylogeny for reference. A number of species icons were obtained from the PhyloPic database (Gearty & Jones 2023). The code to recreate the plot can be found in Supplementary file S3 (see Supplementary material).

The results of the phylogenetic signal analysis revealed that the species considered in the 30 selected studies were more closely related to each other than would be expected by chance (Pagel’s λ = 0.43; *P* < 0.001, see Supplementary file S3 [Supplementary material]). This was potentially driven by the inclusion of multiple species from relatively small families, such as Salmonidae (salmonids) and Catostomidae (suckers) (see Figure 2). It should be noted, however, that it is possible that the sampling of species for the tree may have affected this estimate, given that only a small fraction of all existing fish species are included (roughly 2,000 out of a total of around 30,000 species; Carrete Vega & Wiens 2012).

Out of the 30 studies, 22 explicitly presented data for sexually mature individuals, and 14 explicitly presented data on individuals before they reached sexual maturity.

### Collection methods and causal factors considered

The most common method of collecting fish was via nets. For example, among the 30 selected studies, 15 used various types of nets (e.g. seine nets, gillnets and trawling) to drag or entangle fish, and four used traps sometimes consisting of nets (e.g. fyke nets). In addition, seven studies used electrofishing, four used angling, and one used spearfishing.

Anthropogenic disturbance (e.g. effluents from industry or oil spills; Lindesjöö & Thulin 1992; Marty *et al.* 1997) was considered in 21 of the 43 estimates (Figure 1), and five studies compared injuries and/or deformities between disturbed and undisturbed sites. All other estimates considered injuries or deformities without attributing them specifically to anthropogenic factors.

## Discussion

The review of published estimates of injuries and deformities in wild fish indicates relatively high rates with, on average, 15% of the studied fish presenting at least one kind of injury or deformity. However, this prevalence estimate was representative of certain species that were overrepresented in the literature.

### Prevalence estimates

As mentioned in the *Introduction*, the average proportion of wild fish that was found here to show injuries and/or deformities (15%) represents a snapshot of the fraction of individuals experiencing them at any point in time. Therefore, it likely underestimates the overall number of fish being affected at least once in their lives, partly because of their increased mortality risk, especially in juveniles (which strongly outnumber adults in most fish species; Hill *et al.* 2021). This is likely to be exacerbated in fish affected by deformities, which may explain their lower prevalence (7%) as compared with that of injured fish (21%). The extent to which mortality may have affected prevalence estimates is, however, difficult to know precisely, as only two of the considered studies were found to report mortality rates in fish affected by injuries or deformities (Schneider *et al.* 1996, Homola *et al.* 2014), and only a minority of studies were conducted on juvenile fish. Moreover, the prevalence estimate found here might also be overestimated by the fact that the studies considered for this review likely focused on populations that were suspected *a priori* to present signs of injuries or deformities. Indeed, given that many of the studies were motivated by sudden changes in mortality, apparent disease outbreaks, or high levels of deformities in areas affected by pollution from industries, many of the estimates can likely not be taken as indications of the typical prevalence of injuries and deformities in wild fish and may, therefore, likely overestimate real rates, especially in disturbed environments where prevalence was found to be high. However, this might not apply to cases where fish are sampled simply to obtain a baseline or reference point (see, e.g. Marty *et al.* 1997). Somewhat surprisingly, such baseline estimates of injuries or deformities from non-anthropogenic causes were not uncommon in the present review (Figure 1), probably because they were used as references for comparison with disturbed sites (see Supplementary file S1; Supplementary material).

### Species representation

The fish species that were represented in the studies examining injuries and deformities were found to be more closely related to each other than would be expected by chance. Many of these species are common in aquaculture or have economic value through fisheries (e.g. salmonids; Figure 2). This species bias is unsurprising, given that closely related species will share traits of interest to humans (including how easily they can be accessed and studied). However, from a wild animal welfare perspective, it is potentially unfortunate, as fish of more abundant species but of lower economic value might also experience poor welfare due to injuries or deformities. For instance, fish of low economic value may be the main victims of bycatch, which is likely to be associated with considerable suffering and increased mortality after release (Broadhurst *et al.* 2006). Paradoxically, while several studies have been conducted on the effects of, e.g. trawls on the risk of injury and death in wild fish (see Broadhurst *et al.* 2006 and references within), none seem to focus specifically on the injuries of fish released as bycatch from large fishing operations. From the perspective of fish welfare, this seems like a missed opportunity, given the large number of individuals affected (Mood & Brooke 2024).

### Looking ahead

Future interventions aimed at improving the welfare of wild fish (see e.g. Mueller *et al.* 2017) would benefit from purposely quantifying the prevalence and severity of injuries and deformities in wild fish, as well as from assessing how specific injuries and deformities affect welfare (see, e.g. Weirup *et al.* 2022). Moreover, the examination of injuries and deformities could be carried out in wild fish using more refined methods than the traumatic netting methods used in most of the studies reviewed here. For instance, in the field of conservation, automated detection and classification of fish species have been achieved using footage from underwater cameras, such as stationary cameras, autonomous underwater vehicles (AUVs), or remotely operated vehicles (ROVs) (Marini *et al.* 2018; Ditria *et al.* 2021; Connolly *et al.* 2022). It is also important to note that while tools are currently available for automatically identifying fish from non-fish objects (Ditria *et al.* 2020), and for determining species identity (Villon *et al.* 2018), there are currently no tools available for automatically classifying presence/absence of injuries and deformities. There has been some work on developing tools for detecting welfare-related traits in aquaculture (Barreto *et al.* 2022; Gupta *et al.* 2022), but additional effort is likely required to expand these for use in the wild.

### Animal welfare implications

While the presence of externally visible injuries or deformities is likely indicative of poor welfare, the lack of such injuries or deformities does not necessarily indicate good welfare, given the numerous ways in which negative welfare states can be caused beyond injuries and deformities. There may be many factors causing poor welfare that are not visible from an external examination of injuries or deformities alone, such as the presence of internal parasites (Sterud *et al.* 2007), low oxygen levels, high levels of nitrogenous compounds in the water (MacIntyre *et al.* 2008; Zhou *et al.* 2023), social isolation (Bailey & Moore 2018) or fear of predators (Zanette & Clinchy 2019; Siewert *et al.* 2024). In other words, assessing whether an animal has poor welfare using only injuries or deformities as a tool, is likely associated with relatively good Positive Predictive Value (PPV), i.e. their presence is indicative of poor welfare, but relatively poor Negative Predictive Value (NPV), i.e. their absence is not necessarily indicative of good welfare. While attempting to measure welfare directly would likely be the most helpful, data on fitness-related traits like body condition, feeding success and survival can be useful as well. Several studies have looked at such traits in fish with injuries or deformities, where, crucially, only some of the studies and investigated traits indicate negative welfare-related effects (Sato 2006; Arbuatti *et al.* 2013; Yamamoto *et al.* 2013; Sylvia *et al.* 2023). Additional research to elucidate which conditions result in strong welfare effects, and which cause a negligible loss of welfare, would be useful here.

While assessments of externally visible injuries or deformities may allow for non-disruptive, relatively cheap, and scalable estimates of welfare in wild fish, in most contexts, it will only provide a partial view of wild fish welfare. To determine the contexts where such measures are particularly useful indicators of welfare, validation against more demanding but potentially more reliable methods (e.g. cognitive bias tests) will be highly useful (Rogers *et al.* 2020; Jarvis *et al.* 2021; Browning 2022, Alvarado *et al.* 2025). Other non-disruptive methods for assessing the welfare of wild fish could also be used at the population level, such as screening for pathogens in eDNA (Chapman *et al.* 2021), or estimating mortality rates due to injuries using carcase recovery data (DeWeber *et al.* 2017). Indeed, mortality rates can be indicative of welfare under certain conditions (Ellis *et al.* 2012), such as mortality from starvation (China & Holzman 2014) or parasitism (Lester 1984; Vollset *et al.* 2024). As such, estimates of differences in mortality rates and their causes (see, e.g. Krause *et al.* 2017; Stige *et al.* 2019; Weinz *et al.* 2020) could be particularly useful as a complement to assessing the welfare effects of injuries and deformities, helping identify particularly severe welfare threats.

## Conclusion

Many individual fish are likely to suffer from injuries and deformities in the wild, and this review offers a first glimpse of such threats to wild fish welfare. However, to provide a more accurate picture, these preliminary results would need to be complemented by further empirical studies looking specifically at these aspects across a large variety of fish species, ideally beyond just charismatic and economically valuable species. Moreover, understanding the effects that injuries and deformities may have on the welfare of wild fish would require triangulating these external indicators with other measures, including individual-based high-quality welfare indicators and population level indicators like mortality rates.

## Supporting information

10.1017/awf.2026.10083.sm001 Eckerström Liedholm supplementary material Eckerström Liedholm supplementary material

## Supplementary material

The supplementary material for this article can be found at http://doi.org/10.1017/awf.2026.10083.
